# Depicting deterministic variables within directed acyclic graphs: an aid for identifying and interpreting causal effects involving derived variables and compositional data

**DOI:** 10.1093/aje/kwae153

**Published:** 2024-06-24

**Authors:** Laurie Berrie, Kellyn F Arnold, Georgia D Tomova, Mark S Gilthorpe, Peter W G Tennant

**Affiliations:** School of GeoSciences, College of Science and Engineering, University of Edinburgh, Edinburgh LS2 9NL, United Kingdom; Leeds Institute for Data Analytics, University of Leeds, Leeds LS2 9NL, United Kingdom; Leeds Institute for Data Analytics, University of Leeds, Leeds LS2 9NL, United Kingdom; School of Medicine, Faculty of Medicine and Health, University of Leeds, Leeds LS2 9JT, United Kingdom; The Alan Turing Institute, British Library, London NW1 2DB, United Kingdom; Obesity Institute, Leeds Beckett University, Leeds LS1 3HE, United Kingdom; Leeds Institute for Data Analytics, University of Leeds, Leeds LS2 9NL, United Kingdom; School of Medicine, Faculty of Medicine and Health, University of Leeds, Leeds LS2 9JT, United Kingdom; The Alan Turing Institute, British Library, London NW1 2DB, United Kingdom; Obesity Institute, Leeds Beckett University, Leeds LS1 3HE, United Kingdom

**Keywords:** causal inference, directed acyclic graphs, compositional data, derived variables, composite variables, tautological associations

## Abstract

Deterministic variables are variables that are functionally determined by one or more parent variables. They commonly arise when a variable has been functionally created from one or more parent variables, as with derived variables, and in compositional data, where the “whole” variable is determined from its “parts.” This article introduces how deterministic variables may be depicted within directed acyclic graphs (DAGs) to help with identifying and interpreting causal effects involving derived variables and/or compositional data. We propose a 2-step approach in which all variables are initially considered, and a choice is made as to whether to focus on the deterministic variable or its determining parents. Depicting deterministic variables within DAGs brings several benefits. It is easier to identify and avoid misinterpreting tautological associations, that is, self-fulfilling associations between deterministic variables and their parents, or between sibling variables with shared parents. In compositional data, it is easier to understand the consequences of conditioning on the “whole” variable and to correctly identify total and relative causal effects. For derived variables, it encourages greater consideration of the target estimand and greater scrutiny of the consistency and exchangeability assumptions. DAGs with deterministic variables are a useful aid for planning and interpreting analyses involving derived variables and/or compositional data.

## Introduction

Causal directed acyclic graphs (DAGs) are increasingly popular aids for identifying and estimating causal effects^1^^,^^2^ and for recognizing and understanding various forms of error, bias, and noncausal associations.^3^^‑^^7^ However, little attention has been given to their utility for understanding analyses involving deterministic variables.^8^^,^^9^ A deterministic variable is a variable that is functionally determined by one or more other variables such that its value can be known with certainty once its parents are known.^8^^,^^9^ They are extremely common in health and social science, typically arising in the following types and settings:

1. *Derived variables*. Derived variables are variables that have been functionally created from one or more parent variables.^10^ They include simple derived variables (eg, macrosomia), which are created from a single parent variable (eg, birth weight), and composite derived variables (eg, waist:hip ratio), which are created from 2 or more parent variables (eg, waist circumference and hip circumference).^11^
2. *Compositional data*. Compositional data are a form of hierarchical data that contain “part variables” (eg, fat mass and fat-free mass) that perfectly sum to a “whole” variable (eg, total mass) or a constant.^12^^‑^^14^

Because DAGs are primarily used to consider probabilistic relationships,^15^ deterministic variables have received limited attention within DAGs. Indeed, DAGs containing deterministic variables have additional statistical implications that make them incompatible with many routine causal identification and discovery algorithms.^16^ However, depicting deterministic variables within DAGs can be useful for understanding certain challenges involved in the analyses and interpretation of deterministic variables.

In this paper, we introduce how deterministic variables can be depicted within DAGs and discuss the benefits for identifying and interpreting causal effects involving derived variables and/or compositional data.

## Depicting deterministic variables within DAGs

**Figure 1:**
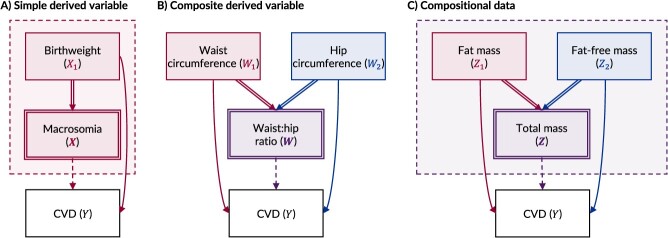
Causal directed acyclic graphs using deterministic notation to depict a simple derived variable (A), a composite derived variable (B), and compositional data (C). Fully determined child variables are represented by double-outlined nodes; deterministic relationships are represented by double-lined arcs; arcs leaving a deterministic variable are represented as dashed arcs; and situations where a child variable and its determining parent variables occur simultaneously in time are enclosed within a dashed-outline box. An example outcome, cardiovascular disease (CVD), has been added to all examples. In panel A, the simple derived variable, macrosomia ($\boldsymbol{X}$), is a binary variable that is fully determined by birth weight (${X}_1$). In panel B, the composite derived variable, waist:hip ratio ($\boldsymbol{W}$), is fully determined by dividing the waist circumference (${W}_1$) by the hip circumference (${W}_2$). In panel C, the “whole” variable, total mass ($\boldsymbol{Z}$), comprises 2 “part” variables, fat mass (${\mathrm{Z}}_1$) and fat-free mass (${\mathrm{Z}}_2$), and can therefore be fully determined by summing both parent components.

A causal DAG is a graphical representation of the hypothesized causal relationships between a set of variables (or “nodes”).^1^^,^^2^ Any 2 variables in the graph may be connected by a unidirectional arrow (or “arc”), which signifies that the first variable (the “parent” or “ancestor”) exerts a causal effect on the second (the “child” or “descendent”). Because a DAG is acyclic, no variable may cause itself at the same moment in time. To ensure that deterministic variables are handled appropriately within DAGs, they should be distinctively depicted. To achieve this, we follow the convention that any “child” variable that is fully determined by one or more “parent” variables is depicted with a double-outlined node.^17^ We also suggest that (1) all arcs entering a deterministic variable should be double-lined, to denote that they are part of a functional, not probabilistic, relationship,^12^ and (2) all arcs leaving a deterministic variable should be dashed; this denotes that, while it may be useful to conceptualize the “implied” causal effect of a deterministic variable, no residual effect exists beyond that caused by the parent variables.^10^^,^^18^ Finally, where a child variable and all determining parent variables occur concurrently, we suggest enclosing the family within a dashed-outline box.^12^ Examples of this notation are given in Figure 1, which depicts a simple derived variable (Figure 1A), a composite derived variable (Figure 1B), and compositional data (Figure 1C).

## Algorithmic approaches and the benefit of DAGS

For many years, deterministic variables were not strictly compatible with DAGs because deterministic variables bring additional statistical dependencies.^1^ This was resolved with the introduction of the *D*-separation criterion (note the uppercase “*D*”), which extends the familiar *d*-separation criterion to accommodate the behavior of deterministic variables.^17^ Despite this, most causal modeling and discovery algorithms are not natively compatible with deterministic variables.^16^ Such variables are hence usually treated as nuisance nodes that need to be identified and removed.^17^ Shachter’s deterministic node reduction algorithm achieves this by identifying all deterministic variables within a DAG and transferring the incoming and outgoing probabilistic arcs to their parent nodes to create barren nodes that may be removed from the graph without losing information about the relationships between the remaining variables.^8^

Simply identifying and removing deterministic nodes is not especially useful when a deterministic variable is the exposure or outcome of interest. In these circumstances, we advocate an alternative 2-step approach to ensure that the assumptions and implications are fully considered. First, a “full” DAG is drawn that includes the deterministic exposure and/or outcome, as well as all determining parents. Next, an explicit choice is made as to whether to focus on the deterministic variable(s) or the determining parents.

## Understanding tautological associations

Perhaps the most straightforward benefit of depicting deterministic variables within DAGs is the ability to identify and avoid misinterpreting tautological associations. We define tautological associations as the self-fulfilling associations that arise when a deterministic variable is analyzed in direct relation to one of its parent variables, or a sibling variable with at least 1 shared parent component.

**Figure 2:**
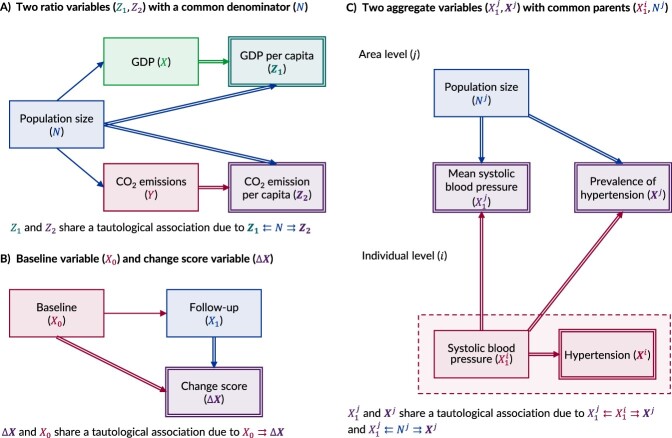
Causal directed acyclic graphs of 3 tautological associations: associations between 2 ratio variables with a common denominator parent variable (A); associations between a change score variable and its baseline parent variable (B); and associations between 2 aggregate variables (C). In panel A, the observed variable’s gross domestic product (GDP) ($\boldsymbol{X}$) and carbon dioxide (CO_2_) emissions ($\boldsymbol{Y}$) are both caused by population size ($N$), making $N$ a confounder for the apparent relationship between $X$ and $Y$. Two composite derived variables have also been created by dividing both $X$ and $Y$ by $N$ to create GDP per capita (${\boldsymbol{Z}}_{\mathbf{1}}$) and CO_2_ emissions per capita (${\boldsymbol{Z}}_{\mathbf{2}}$), respectively. Since both ${\boldsymbol{Z}}_{\mathbf{1}}$ and ${\boldsymbol{Z}}_{\mathbf{2}}$ share the same parent variable ($N$), they will share a tautological association. In panel B, the baseline measurement of a repeated measure variable (${X}_0$) causes the follow-up measurement (${X}_1$), from which a change score variable ($\boldsymbol{\Delta} \boldsymbol{X}$) has been created by subtracting ${X}_0$ from ${X}_1$. Since ${X}_0$ is a deterministic parent of $\boldsymbol{\Delta} \boldsymbol{X}$, they share a tautological association. In panel C, where the subscript $i$ denotes the individual level and $j$ denotes the area level, a simple derived variable, hypertension (${\boldsymbol{X}}^{\boldsymbol{i}}$), is a dichotomized individual-level variable that is fully determined by the continuous individual-level variable systolic blood pressure (${\boldsymbol{X}}_{\mathbf{1}}^{\boldsymbol{i}}$). Area-level mean systolic blood pressure (${\boldsymbol{X}}_{\mathbf{1}}^{\boldsymbol{j}}$) and area-level prevalence of hypertension (${\boldsymbol{X}}^{\boldsymbol{j}}$) are determined at the aggregate level from ${\boldsymbol{X}}_{\mathbf{1}}^{\boldsymbol{i}}$ and the area-level population (${\boldsymbol{N}}^{\boldsymbol{j}}$). Since both ${\boldsymbol{X}}_{\mathbf{1}}^{\boldsymbol{j}}$ and ${\boldsymbol{X}}^{\boldsymbol{j}}$ share the same two parent variables (${\boldsymbol{X}}_{\mathbf{1}}^{\boldsymbol{i}},{\boldsymbol{N}}^{\boldsymbol{j}}$), they share a tautological association.

The problem of tautological associations was first identified by Karl Pearson in 1897 in the context of analyzing ratio variables.^19^ Ratio variables (eg, $X/N$) are composite derived variables created by dividing one parent variable (eg, $X$) by a second parent variable (eg, $N$).^20^ Assuming faithfulness,^1^ Pearson warned that 2 ratio variables with a shared denominator parent variable (eg, $X/N$ and $Y/N$) would share a “spurious (organic) correlation” even if the numerators (eg, $X$, $Y$) are unrelated.^19^^(p.490)^ Using deterministic notation, this phenomenon can be depicted and understood using a DAG that contains the 3 parent variables (ie, $X$, $Y$, and $N$) and the 2 child variables ($X/N$ and $Y/N$) (Figure 2A).

Perhaps the most well-known example of a tautological association occurs in the context of analyzing change score variables. Change score variables (eg, $\Delta X={X}_1-{X}_0$) are composite derived variables created by subtracting an earlier measure of a time-varying variable (eg, ${X}_0$) from a subsequent measure of that variable (eg, ${X}_1$) (Figure 2B). In 1962, Oldham warned that change score variables share a negative “spurious correlation” with their baseline parent variable that is “entirely produced by our arithmetical procedure.”^21^^(p.970)^ Known sometimes as the “law of initial value,” this occurs because of the negative parameterization of the baseline variable in the change score variable.^22^ Other examples of tautological associations can be found in the literature, under the term “mathematic(al) coupling,”^23^ although most examples probably occur in applied analyses with no awareness of the phenomenon.

In statistical terms, tautological associations are neither erroneous nor biased.^24^ The expected association between 2 ratio variables, for example, is an accurate reflection of their common denominator variable.^24^ However, for causal interpretation, inferential bias can occur when the underlying tautology is not recognized and the resulting associations are misattributed to other (causal) mechanisms. Such misinterpretations are probably more common for composite derived variables with many parent variables, since the deterministic origins become easier to overlook. Nevertheless, there are examples of simple tautological associations (eg, between hypertension and blood pressure) being overlooked when analyses are conducted at an aggregate level (Figure 2C).^25^ By placing the parent variables for all deterministic exposure and outcome variables within a DAG, we believe such mistakes become less likely.

## Considering simple derived variables

Most simple derived variables are created for statistical rather than causal reasons. For example, an exposure variable may be log-transformed to more accurately model a nonlinear relationship with the outcome.^26^ Although important for estimation,^27^ such transformations have limited implications for causal reasoning, except where the transformation leads to a loss of information—for example, due to coarsening.^28^

Coarsening commonly occurs when a dichotomous variable (eg, smoker/nonsmoker) is created by collapsing a continuous or multinomial variable (eg, number of cigarettes smoked per day) into 2 categories.^29^ Coarsening the exposure has particular implications for the consistency assumption, which requires that there must be no two versions of the exposure such that, for the same exposure value, the different versions have different probabilities of one or more possible outcomes.^30^ If multiple versions of the exposure are collapsed into a single value, these versions need to have the same effect on the outcome to provide a well-defined causal effect—an assumption known as effect equivalence.^28^ Without effect equivalence, the estimated effect of the coarsened variable will be a poorly defined weighted average of the effects of the multiple parent versions of the exposure and their frequencies in the study population.^31^

Coarsening the exposure can be especially problematic for instrumental-variable analyses, because a coarsened exposure will probably violate the exclusion restriction assumption—that is, that the instrument has no effect on the outcome other than through the exposure.^32^^,^^33^ This is because the instrument is likely to cause the outcome through variation in the parent exposure variable that is not captured by the coarsened child exposure.^32^^,^^33^

**Figure 3:**
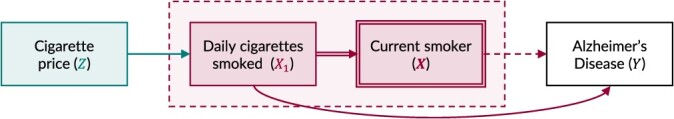
Causal directed acyclic graph of an instrumental-variable scenario with a coarsened exposure variable. In this scenario, the continuous variable, number of cigarettes smoked per day (${X}_1$), has been coarsened into a dichotomized variable, current smoker/nonsmoker ($\boldsymbol{X}$). The implied causal effect of $\boldsymbol{X}$ on the outcome, Alzheimer disease (${Y}$), is depicted with a dashed arc, although this effect is technically entirely explained by ${X}_1$. Another direct path is depicted from the parent exposure to the outcome (${X}_1\to Y$), which represents the residual (dose–response) effect of ${X}_1$ on the outcome that does not act through $\boldsymbol{X}$. For the causal effect of $\boldsymbol{X}$ on ${Y}$ to be robustly estimated, this residual path ${X}_1\to Y$ must be zero (the effect equivalence assumption). This is apparent if we imagine estimating the effect of $\boldsymbol{X}$ on ${Y}$ using an instrumental variable, cigarette price ($Z$), since the residual path ${X}_1\to Y$, if nonzero, would violate the exclusion restriction assumption.

To illustrate, suppose we are interested in the average causal effect of smoking on the risk of Alzheimer disease. Smoking is a dichotomized child of daily number of cigarettes smoked. If the number of cigarettes smoked per day has a dose–response relationship with the risk of Alzheimer disease, the average causal effect of smoking will reflect a poorly defined weighted average of different smoking levels. If we tried to estimate this effect using an instrumental variable (eg, cigarette price), the exclusion restriction assumption would be violated by any such dose–response effect.^32^^,^^33^ This assumption can be seen visually in a DAG containing both the parent and child exposure variables as the (residual) path between the parent exposure and the outcome (Figure 3).

Although the issues with variable coarsening cannot be solved by simply depicting derived variables and their parent variables within DAGs, the practice may help to ensure that the resulting implications and assumptions are more explicitly considered.

## Considering compositional data

The benefits of depicting deterministic variables within DAGs increase with the complexity of the variables and/or relationships being considered. Compositional data is a common form of complex data structure that naturally contains deterministic relationships because the “part” variables sum to a “whole” variable or constant (Figure 1).^12^^,^^14^ This makes compositional data notoriously challenging to analyze and interpret correctly.^12^^,^^14^^,^^34^^,^^35^ Pearson’s warning on the use of ratio variables was allegedly motivated by observing biologists dividing bone measurement variables (eg, femur length) by length measurement variables (eg, leg length).^19^ Since then, the area of compositional data analysis (CoDA) has emerged to develop specific analytical strategies.^13^^,^^14^ We focus on the insights that arise from considering compositional data within DAGs.^12^^,^^34^^,^^35^

### Simplifying features of compositional data

There are 2 important features of compositional data that reduce the potential complications when compared with composite derived variables. First, the “whole” variable can usually be directly observed. Indeed, whether a variable is a “whole” or a “part” is often a matter of perspective or convenience rather than external structure. All variables can potentially be divided into further parts or summed to a greater whole. The choice of whether to focus on the “whole” or the “parts” is therefore usually a trade-off between the competing benefits of aggregation and subdivision.

The second key feature of compositional data is that the “whole” variable and all of the “part” variables occur at the same moment in time. This avoids many of the more serious issues affecting composite derived variables discussed below.

### Choosing the target estimand

Analyses of compositional data generally consider 2 types of estimands: total compositional effects and relative compositional effects. Total compositional effects represent the effects of increasing the “whole” variable either by intervening on the “whole” directly or through one or more specified “part” exposures.^12^^,^^34^^‑^^36^ Relative compositional effects represent the joint effect of increasing a specified “part” exposure while simultaneously decreasing one or more substituting “parts” to keep the “whole” fixed.^12^^,^^34^^‑^^36^ Different analytical strategies are required to estimate these two effects, and misinterpretations occur when the wrong strategy is used inadvertently, or when the relative nature of structurally fixed data (eg, time-use data) is not recognized.^12^^,^^34^^,^^35^

Since compositional data occur at the same time, the “whole” and “part” variables may be drawn in multiple ways, but it is intuitive to consider the “whole” as being determined by the “parts” (Figure 1C).^12^ Drawn like this, the “whole” can be usefully interpreted as a *collider* for the “parts,” and it is clear that conditioning on the “whole” introduces a dependency between the “parts.”^12^ Therefore, the individual effect of a specific “part” cannot be estimated when conditioning on the “whole.”^12^

**Figure 4:**
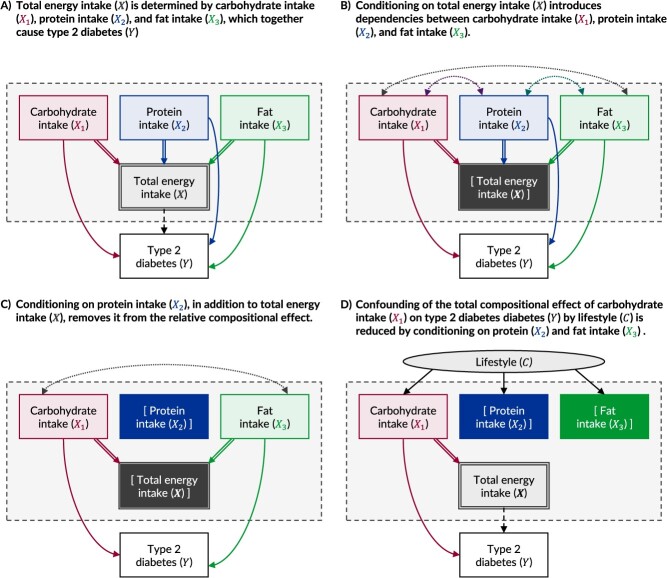
Causal directed acyclic graphs (DAGs) examining the identification of causal effects in compositional data, here represented by total energy intake. The “full” DAG for the scenario is depicted in panel A, where the “whole” variable, total energy intake ($\boldsymbol{X}$), is fully determined by intake from 3 “part” variables—carbohydrate intake (${X}_1$), protein intake (${X}_2$), and fat intak*e* (${X}_3$)—which together cause type 2 diabetes (${Y}$). In panel B, the “whole” has been conditioned on (denoted by square brackets and an absence of onward arcs), inducing conditional dependencies between the unconditioned “part” variables. The causal effect of any “part” variable (eg, ${X}_1$) on ${Y}$ would thus be relative to the other unconditioned “part” variables (eg, ${X}_2$, and ${X}_3$). In panel C, one of the other “part” variables (${X}_2$) has been conditioned on in addition to the whole, which removes it from the relative compositional effect; the effect of ${X}_1$ on ${Y}$ will thus be relative to ${X}_3$ only. In panel D, a confounding variable, lifestyle (${C}$), is introduced that commonly causes all “part” variables. Confounding from such common causes can be reduced by conditioning on other “part” variables to block the confounding paths downstream.

To illustrate, we consider the total effect of carbohydrate consumption on the risk of diabetes, where the consumption of carbohydrates, proteins, and fats determines the total energy intake (Figure 4A). In nutritional epidemiology, it is common to evaluate the effects of one or more specific dietary component(s) on subsequent health outcomes while conditioning on total energy intake as a proxy “confounder” for the diet.^37^ However, when drawn as suggested, it is apparent that conditioning on total energy intake would introduce a dependency between carbohydrate consumption and the other macronutrient variables, creating a relative effect (Figure 4B). Exactly *which* relative effect will depend on whether additional adjustments are made for any of the other macronutrients (Figure 4C).^34^^,^^35^

### Identifying and estimating causal effects in compositional data

This simple 3-nutrient example demonstrates how retaining both the “whole” and “part” variables within a DAG can help with understanding compositional data. The optimal analytical strategy then depends on whether the investigator is interested in the total effect of a particular “part,” a relative effect of a particular “part,” or the summary effect of the “whole.” There are, however, additional caveats.

First, the exchangeability assumption requires that the units of analysis have an equal probability of all possible values of the outcome at the time of exposure; that is, there must be no confounding or selection bias for the exposure–outcome relationship of interest.^1^^,^^3^ In compositional data, confounding can arise from common causes of the “parts,” even if these causes have no residual effect on the outcome, because each part is itself likely to cause the outcome. In our 3-nutrient example, such common causes might include lifestyle behaviors. Ideally, these common causes should be directly measured and conditioned on, but this is not always possible. Instead, confounding by common causes may be reduced by conditioning on other “parts” to block the confounding paths downstream (Figure 4D). Where each “part” has a unique effect and variance, this requires measuring and conditioning on every “part” variable. In practice, aggregated “part” variables are often used, such as “remaining energy intake” (ie, energy from all parts *except* the exposure), but this may introduce residual confounding wherever the causal effect of each “part” differs from the average effect of the aggregate variable.^34^^,^^35^

In some situations, the average effect of increasing the “whole” may be of more interest than the individual “parts” specifically. Here, it may be reasonable to discard the parent variables from the DAG and treat the “whole” variable as the exposure. However, this increases the chance of violating the consistency assumption, since the same value of the “whole” can be obtained from many different combinations of the “parts.” If each “part” has different causal effects on the outcome and/or different variances, the summary effect of the “whole” will not be the mean-weighted average effect but will be distorted towards those “parts” with the largest variances^38^; we have previously termed this phenomenon *composite variable bias*.^34^^,^^35^

In theory, measuring and modeling all components offers the ideal approach to compositional data analyses. In practice, the benefits of achieving greater consistency need balancing against the demands of modeling ever more variables. As the number of components increases, there is a greater chance of violating the positivity assumption, which requires that within every stratum there must be a nonzero probability of all (relevant) values of the exposure being observed.^39^ The choice of whether to focus on the “whole” or the “parts” will therefore involve balancing the desired degree of consistency with the quality and availability of the data.

### Considering composite derived variables

Within a DAG, a composite derived variable appears similar to a “whole” variable in compositional data, with 2 or more “parents” causing a fully determined “child” (Figure 1). However, there are some important features of composite derived variables that make them particularly challenging for causal effect estimation. First, many composite derived variables cannot be directly measured; they can only be known once the parents themselves are known.^10^ Second, the parent variables may be subject to a range of functional transformations besides addition, including subtraction, division, and exponentiation.^11^ Finally, the parent components may not occur at the same moment in time, giving them different temporal positions within the DAG. It is therefore extremely important to consider the nature and purpose of every composite derived variable being considered as a potential exposure or outcome.

### Choosing the target estimand

Composite derived variables are commonly constructed for one of 2 reasons:

1. To *summarize* several interrelated variables (eg, deprivation index) into a single variable, either to capture a latent concept (eg, socioeconomic circumstances), to define a multifactorial state (eg, metabolic syndrome), or to provide a global summary (eg, disease activity scores).

2. To *standardize* one or more variables against one or more other variables (eg, gross domestic product per capita), either to account for another variable (eg, population size) or to rescale to a common unit (eg, percentage change).

Whether a composite variable has been constructed to summarize or to standardize has immediate implications for its analysis and/or interpretation. The creation of a summary variable implies an interest in estimating the average effect of, or on, the parent variables. Conversely, composite variables that seek to standardize imply an interest in one or more parent variables while controlling for one or more “nuisance” components. Ratio variables and change score variables, for example, both seek to isolate one parent from another, using division and subtraction, respectively.^19^^,^^22^^,^^24^ Unfortunately, such approaches simply transform, rather than remove, the nuisance components.^19^^,^^22^^,^^24^^,^^34^ Causal analyses of ratio variables and change score variables are hence particularly prone to inferential bias.^19^^,^^22^^,^^24^^,^^34^ For most standardized composite variables, it is likely that the true target of interest is one or more target parent variables *conditional* on one or more nuisance parent variables. Rather than attempting an algebraic solution, such conditioning should be attempted using an appropriate approach, such as covariate adjustment within a linear regression model.

**Figure 5:**
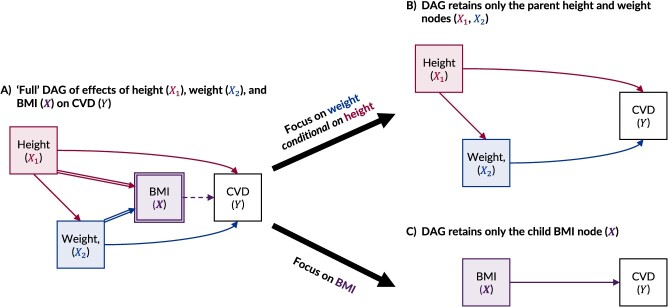
Causal directed acyclic graphs (DAGs) considering the causal effect of a composite derived variable (body mass index, calculated as weight [kg]/height [m]^2^; BMI) on an outcome (cardiovascular disease [CVD]). The “full” DAG in panel A shows BMI ($\boldsymbol{X}$) as a fully determined child of height (${X}_1$) and weight (${X}_2$). An explicit choice can be made, based on the target estimand of interest, to either retain and focus on the parent nodes ${X}_1$ and ${X}_2$, as shown in panel B, or retain and focus on the child node $\boldsymbol{X}$, as shown in panel C.

To illustrate, we consider the causal effect of body mass index (BMI; weight [kg]/height [m]^2^) on the risk of cardiovascular disease (Figure 5A). In probabilistic terms, BMI contributes no information beyond what is captured by weight and height.^18^ Deterministic node reduction would hence reduce BMI to a barren node that may be removed without losing information about the relationship between the remaining variables (Figure 5B). This explains previous assertions that “no causal knowledge is gained by estimating a nonexistent effect of body mass index”.^18^^(p.957)^ Nevertheless, BMI may still have some utility depending on the target estimand and our reasons for creating the composite derived variable.

Since BMI is constructed by dividing weight by height squared, it seems reasonable to assume it was conceived to standardize weight by height. However, inventor Adolphe Quételet (1832) offers no specific motivation beyond reporting that “weight increases approximately with the square of the height.”^40^^(p.28)^ Similarly, Keys et al,^41^ who transformed the name and prominence of the index, only appeared interested in finding the best proxy measure of skinfold thickness. Therefore, whether BMI is intended purely as a measure of weight standardized for height or as a summary of information about both weight and height cannot be known from history or algorithm, but the two perspectives carry different implications. If BMI is hypothesized as a valuable joint summary of weight and 1/height^2^, then focusing on the composite measure may be reasonable, notwithstanding the issues discussed below (Figure 5C). Alternatively, if BMI is simply a measure of weight standardized by height, the appropriate target would be *weight conditional on height* (Figure 5B). Since the two approaches likely provide different results, determining the true estimand of interest is clearly extremely important.

### Identifying and estimating causal effects involving composite variables

Regardless of their potential utility, most composite derived variables are likely to experience issues with satisfying the consistency and exchangeability assumptions. As with “whole” variables in compositional data, composite derived variables have an inherent risk of consistency violations, because the same value can be obtained from many different combinations of the parents. However, since composite derived variables are typically made from a more heterogeneous mix of parent variables than the “whole” variable in compositional data, the impact of these violations may be more severe. When only the summary effect of the composite is available, the individual parent effects are lost and it becomes impossible to know which parent variables are responsible and to what extent.^42^ Furthermore, due to composite variable bias, the summary effect of the composite derived variable will be skewed towards the parent variables with the largest variation within the sample,^38^ which may lead to sample-specific effects that do not transport reliably.^31^^,^^43^

Regardless, the exchangeability assumption is likely the greatest barrier to identifying the causal effect of, or on, a composite derived variable. In theory, robustly identifying the causal effect of, or on, a composite derived variable requires that all confounding and selection paths be closed for all parent variables. Unfortunately, when working with the composite parent variable alone, the unique paths to and from each parent variable become conflated. Attempts to block confounding paths may therefore suffer residual confounding, since only the diluted summary effect is modeled. More concerningly, if the parent variables occur at different moments in time, it is possible they will have different relationships with the supposed confounders. Indeed, it is possible that a confounder for one parent variable may be a mediator for another, leaving no means to appropriately adjust for confounding without also blocking part of the true causal effect. Identifying the causal effect of a composite derived variable, as with any analysis of multiple exposures, therefore requires no time-varying confounding.^44^

**Figure 6:**
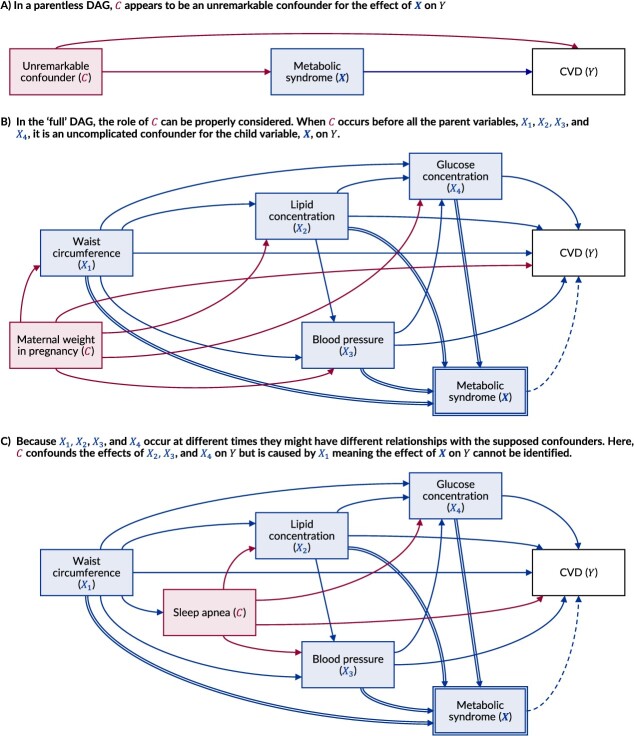
Causal directed acyclic graphs (DAGs) examining the challenges of identifying a causal effect for a composite derived variable (metabolic syndrome) when the parent variables are separated in time. When a composite derived variable, such as metabolic syndrome ($\boldsymbol{X}$), is included in a DAG without including the parents, the distinct relationship with all other contextual variables may be overlooked. In panel A, the contextual variable ${C}$ appears to be an unremarkable confounder for the effect of $\boldsymbol{X}$ on the outcome, risk of cardiovascular disease (CVD) ($Y$), because the parents of $\boldsymbol{X}$ have not been considered. In fact, ${C}$ may have very different relationships with the parent variables—waist circumference (${X}_1$), blood lipid concentration (${X}_2$), blood pressure (${X}_3$), and glucose concentration (${X}_4$)—because they do not occur at the same point in time. In panel B, the variable ${C}$ (eg, maternal weight in pregnancy) occurs before all of the parent components, making it an uncomplicated confounder of $\boldsymbol{X}$. In panel C, the variable ${C}$ (eg, sleep apnea) occurs in between the variables that make up metabolic syndrome; it is caused by ${X}_1$ but causes ${X}_2$, ${X}_3$, and ${X}_4$, meaning it simultaneously confounds and mediates different parts of the effect of $\boldsymbol{X}$ on ${Y}$, making this effect impossible to identify.

 To illustrate, we consider the causal effect of metabolic syndrome on the risk of cardiovascular disease (Figure 6). Metabolic syndrome is a composite derived variable, commonly studied as an exposure, created from waist circumference, lipid concentration, blood pressure, and glucose concentration. In a parentless DAG, we might draw the relationship between metabolic syndrome and cardiovascular disease as shown in Figure 6A. With metabolic syndrome depicted as a single node in time, the role of other contextual variables, represented by $C$, may seem unremarkable (Figure 6A). However, the parent variables of metabolic syndrome are unlikely to occur at the same time; instead, it is likely that some of the parents (eg, waist circumference) may cause some of the other parents (eg, glucose concentration). A variable $C$ might therefore have a very different relationship with the parent components depending on when it occurred. If $C$ occurred before birth (eg, maternal weight in pregnancy), it would likely cause all the components of metabolic syndrome and be an uncomplicated confounder for its effect on the risk of cardiovascular disease (Figure 6B). However, if $C$ occurred in adulthood (eg, sleep apnea), then it might be caused by some “earlier” parts of metabolic syndrome (eg, waist circumference),^45^ while in turn causing other “later” parts (eg, glucose concentration) (Figure 6C).^46^ Although these specific examples can be debated, the “true” $C$ is likely to represent multiple variables, each of which may have different relationships with the individual components of metabolic syndrome. In this case, the time separation between the different parents of metabolic syndrome therefore makes the causal effect of metabolic syndrome on the risk of cardiovascular disease impossible to identify and estimate. While such issues may sometimes be avoided with repeated measures, we believe the assumption of no time-varying confounding can only be explicitly considered by depicting all parent variables within a DAG.

## Conclusion

Deterministic variables are ubiquitous in health and social science research due to the widespread use of derived variables and the common occurrence of compositional data. Unfortunately, despite repeated warnings over many decades,^19^^,^^21^^‑^^24^^,^^47^ the analytical and interpretational challenges of such variables remain largely underappreciated. With appropriate care and notation, we believe that DAGs can provide a novel and effective means to transform our recognition and understanding of these issues. We therefore encourage researchers to consider including deterministic variables in their DAGs when they are planning and/or interpreting analyses involving derived variables and/or compositional data.

Box 1
**Glossary of terms.**

**Barren nodes**

In a directed acyclic graph, barren nodes are nodes that do not cause any other nodes.^8^

**Change score variables**

Change score variables (also known as difference score variables, gain score variables, or change-from-baseline variables) are a variety of composite derived variable in which an earlier measure of a time-varying variable is subtracted from a subsequent measure of that variable.^22^ For example, gestational weight gain (ie, $\Delta W={W}_1-{W}_0$) is a change score variable made by subtracting a pregnant person’s weight at the start of pregnancy (ie, ${W}_0$) from their weight at the end of pregnancy (ie, ${W}_1$).

**Compositional data**

Compositional data, also known as comparative data, is a form of hierarchical data (formally known as a “mereology”) that contains “part” variables (or “meronyms”) that perfectly sum to a “whole” variable (a “holonym”) or a constant.^12^ For example, the total number of children and the total number of adults (the “part” variables) sum to the total population (the “whole” variable). Alternatively, the total time spent physically active and the total time spent inactive (the “part” variables) sum to a constant (total time in a day).

**Compositional data analysis**

The methodological area focused on the analysis of compositional data, historically with a focus on geometric transformations.^13^^,^^14^

**Composite derived variables**

Composite derived variables, also known simply as composite variables or compound variables, are variables that have been functionally created from 2 or more parent variables.^11^ The value of a composite derived variable can be known with certainty once the values of all parents are known. For example, the Clinical Disease Activity Index is a composite derived variable created by adding together 4 parent variables: the total number of swollen joints, the total number of tender joints, a patient-reported measure of disease severity, and a clinician-reported measure of disease severity.^48^

**Composite variable bias**

Composite variable bias refers to the systematic divergence between the average causal effect of a deterministic variable and the mean-weighted average causal effect of its parent variables. In general, the average effect of the deterministic variable will be distorted towards the components with the largest variance.^38^ For example, consider the causal effect of metabolic syndrome—a composite derived variable created from waist circumference, lipid concentration, blood pressure, and blood glucose concentration—on the risk of cardiovascular disease. If these parent components have different variances and different causal effects on the risk of cardiovascular disease, we can expect that the average causal effect of metabolic syndrome will differ from the mean-weighted average causal effect of the parent components.

**Coarsening**

Coarsening is the process of collapsing a continuous variable or higher-order categorical variable into a lower-order categorical variable, such as a dichotomized variable. A coarsened variable has less information than its parent variable.^28^ Unless the effect equivalence assumption is met, the causal effect of, or on, a coarsened variable may produce a biased estimate of the causal effect of, or on, the parent variable.^28^

***D*-separation**

*D*-separation (with an uppercase “D”) is an extension of the *d*-separation criterion for identifying whether 2 (sets of) variables (eg, $\boldsymbol{X}$, $\boldsymbol{Y}$) are independent conditional on a third set of variables (eg, $\boldsymbol{Z}$) that accounts for the additional dependencies created by deterministic variables.^1^ For $\boldsymbol{X}$ and $\boldsymbol{Y}$ to be independent conditional on $\boldsymbol{Z}$ (ie, D-separated by $\boldsymbol{Z}$), there must be no path between $\boldsymbol{X}$ and $\boldsymbol{Y}$ where (1) all collider nodes on that path are in $\boldsymbol{Z}$ or are descended from $\boldsymbol{Z}$ and (2) all other (noncollider) nodes are outside $\boldsymbol{Z}$ and/or not functionally determined by $\boldsymbol{Z}$.^1^

**Deterministic node reduction**

Deterministic node reduction is an algorithm for reducing the number of nodes in a directed acyclic graph. The algorithm involves identifying all deterministic variables and transferring their incoming and outgoing probabilistic arcs to their parents.^8^ The resulting barren nodes may then be removed from the graph without losing information about the relationship between the remaining variables.^8^

**Derived variable**

Derived variables are variables that have been functionally created from one or more parent variables.^10^ They include simple derived variables and composite derived variables.

**Deterministic variables**

Deterministic variables are variables that are functionally determined by one or more other variables, such that their value can be known with certainty once their parents are known.^8^^,^^9^ Deterministic variables occur in compositional data and when derived variables are created.

**Dichotomized variables**

Dichotomized variables are simple derived variables, in which a continuous or multinomial parent variable has been collapsed into 2 categories.^29^ For example, macrosomia is a dichotomized variable made from dichotomizing birth weight; birth weights of under 4500 g are considered normal and birth weights of 4500 g or more are considered macrosomic.

**Effect equivalence**

The effect equivalence assumption, which applies to the estimation of causal effects involving coarsened variables, requires that all versions of the exposure that have been collapsed into a single value must have the same effect.^28^ This assumption is violated if a residual dose–response relationship exists that is not captured by the coarsened variable.^28^ Without effect equivalence, the estimated effect of the coarsened variable will be a poorly defined weighted average of the effects of the multiple parent versions of the exposure and their frequencies in the study population.^31^ For example, the estimated effect of hypertension on the risk of vascular dementia would violate the effect equivalence assumption if, among persons categorized as having hypertension, those with severe hypertension had a greater risk than those with mild hypertension.

**Ratio variables**

Ratio variables are composite derived variables, in which one parent variable (eg, $X$) is divided by a second parent variable (eg, $N$).^20^ For example, gross domestic product (GDP) per capita is a ratio variable made by dividing total GDP by total population.

**Relative compositional effects**

In compositional data, a relative compositional effect is the joint effect of increasing a specified “part” exposure while simultaneously decreasing one or more substituting “parts” to keep the “whole” fixed.^12^^,^^34^^‑^^36^ For this reason, relative compositional effects are sometimes known as substitution effects.

**Simple derived variables**

Simple derived variables, also known as transformed variables, are variables that have been functionally created from a single parent variable. The value of a simple derived variable can be known with certainty once the value of its parent variable is known. For example, 5-year age group is a simple derived variable that is made by categorizing the continuous age variable into 5-year categories (eg, <4 years, 5-9 years, 10-14 years, etc).

**Tautological associations**

Tautological associations, also known as spurious organic correlations and mathematical coupling, are the self-fulfilling associations that arise when a deterministic variable is analyzed in direct relation to one of its parent variables or to a sibling deterministic variable with at least one shared parent variable.^19^^,^^23^^,^^24^ For example, gestational weight gain is a deterministic variable made by subtracting weight at the end of pregnancy from weight at the start of pregnancy; we can therefore expect gestational weight gain to share a tautological association with both weight at the start of pregnancy and weight at the end of pregnancy. Alternatively, GDP per capita and number of hospital beds per capita are two ratio variables with a common parent variable (total population); we can therefore expect them to share a tautological association.

**Total compositional effects**

In compositional data, a total compositional effect is the effect of increasing the “whole” variable either by intervening on the “whole” directly or through one or more specified “part” exposures.^12^^,^^34^^‑^^36^ Total compositional effects are sometimes known as “additive effects” because they describe the effect of “adding” to the total by increasing one or more parts.

## Data Availability

No original data were used in this paper.
